# B Cells and Tumor Immunometabolism: Emerging Insights into Immune Regulation and Therapeutic Resistance

**DOI:** 10.3390/antib15050087

**Published:** 2026-09-17

**Authors:** Swati Gupta, Sandip Rath, Surathi Maiti, Tapas Das, Farhat Afrin

**Affiliations:** 1 Centre for Interdisciplinary Sciences, JIS Institute of Advanced Studies and Research, JIS School of Medical Science & Research Campus, JIS University, Santragachi, Howrah 711112, West Bengal, India; 2 Biochemistry Department, Tata Medical Centre, 14 Major Arterial Road, Rajarhat, Newtown, Kolkata 700156, West Bengal, India; 3 Department of Microbiology, College of Medicine and Jawaharlal Nehru Memorial Hospital, The West Bengal University of Health Sciences, Kalyani 741235, West Bengal, India; 4 Department of Health & Family Welfare, Revised National Tuberculosis Control Programme (RNTCP), Government of West Bengal, Barasat 700124, West Bengal, India

**Keywords:** tumor microenvironment, regulatory B cells, tumor-infiltrating B cells, tumor immunometabolism, hypoxia, metabolic reprogramming, tertiary lymphoid structures, therapeutic resistance, immune checkpoint blockade, lactate, adenosine, PGE2, GABA

## Abstract

Cancer progression and therapeutic resistance are shaped by reciprocal dynamic interactions between malignant cells and the metabolically altered tumor microenvironment (TME). Tumor-associated hypoxia, glucose and amino acid competition, extracellular acidity, lactate accumulation, adenosine, prostaglandin E2 (PGE2) and other metabolic signals can remodel immune cell function and determine whether inflammation is tumoricidal or tumor-supportive. Although B lymphocytes have traditionally been viewed as antibody-producing cells, tumor-infiltrating B (TIL-B) cells comprise functionally heterogeneous populations that can act as antigen-presenting cells, cytokine and chemokine producers, antibody-secreting cells, cytotoxic effectors, regulatory B cells (Bregs) and organizers of tertiary lymphoid structures (TLSs). Their functional state is strongly influenced by the metabolic and spatial context in which they reside. This review focuses on the intersection of B cell biology and tumor immunometabolism, emphasizing how hypoxia, lactate, nutrient limitation, adenosine, PGE2, kynurenine and B cell-derived γ-aminobutyric acid (GABA) may shape B cell states and their interactions with myeloid and lymphoid cells. We discuss how metabolically conditioned Bregs and immunoglobulin (Ig)A-skewed humoral responses can contribute to immune suppression, whereas metabolically competent antigen-presenting, IgG-biased and TLS-associated B cell responses may support effective anti-tumor immunity. Importantly, the effects are tumor type- and context-dependent: B cell/TLS signatures are associated with favorable outcomes in several breast, lung and other solid tumors, whereas B cell-centered immune landscapes can be suppressed or neutral in pancreatic cancer and IgA-dominated responses may be unfavorable in selected malignancies. We further examine how these states may influence sensitivity or resistance to immune checkpoint blockade, chemotherapy, radiotherapy and cellular therapies. Finally, we highlight B cell metabolic pathways as potential therapeutic entry points and identify priorities for spatial metabolomics and prospective interventional studies.

## 1. Introduction

Cancer is a major cause of morbidity and mortality worldwide and treatment failure frequently reflects not only tumor-intrinsic alterations but also adaptive interactions between malignant cells and their surrounding microenvironment via the circulatory and lymphatic networks [1,2,3]. Surgery, radiotherapy, chemotherapy, targeted therapy and immunotherapy can all be effective. However, the durability of treatment responses is constrained by tumor heterogeneity, immune escape and the capacity of tumor and stromal cells to remodel the local metabolic environment. The tumor microenvironment (TME) is therefore not simply a collection of non-malignant cells but a dynamic niche or ecosystem in which oxygen, glucose, amino acids, lipids, extracellular pH and immunoregulatory metabolites influence the phenotype and function of immune cells [3,4,5].

B lymphocytes are particularly relevant to this ecosystem because they occupy multiple functional states. Following B cell receptor (BCR) engagement and appropriate innate or T cell-derived signals, B cells can proliferate, enter the germinal center (GC), undergo somatic hypermutation and class switching and differentiate into memory B cells or antibody (Ab)-secreting plasma cells (ASCs) [6,7]. Within tumors, however, these canonical pathways are modified by chronic antigen (Ag) exposure, inflammatory mediators and metabolic stress. Consequently, the same broad B cell compartment may contain tumor-promoting regulatory B cells (Bregs), immunoglobulin (Ig)A-producing or immunoregulatory populations as well as Ag-experienced, IgG-biased, tertiary lymphoid structure (TLS)-associated B cells that support anti-tumor immunity [8,9,10].

**Figure 1:**
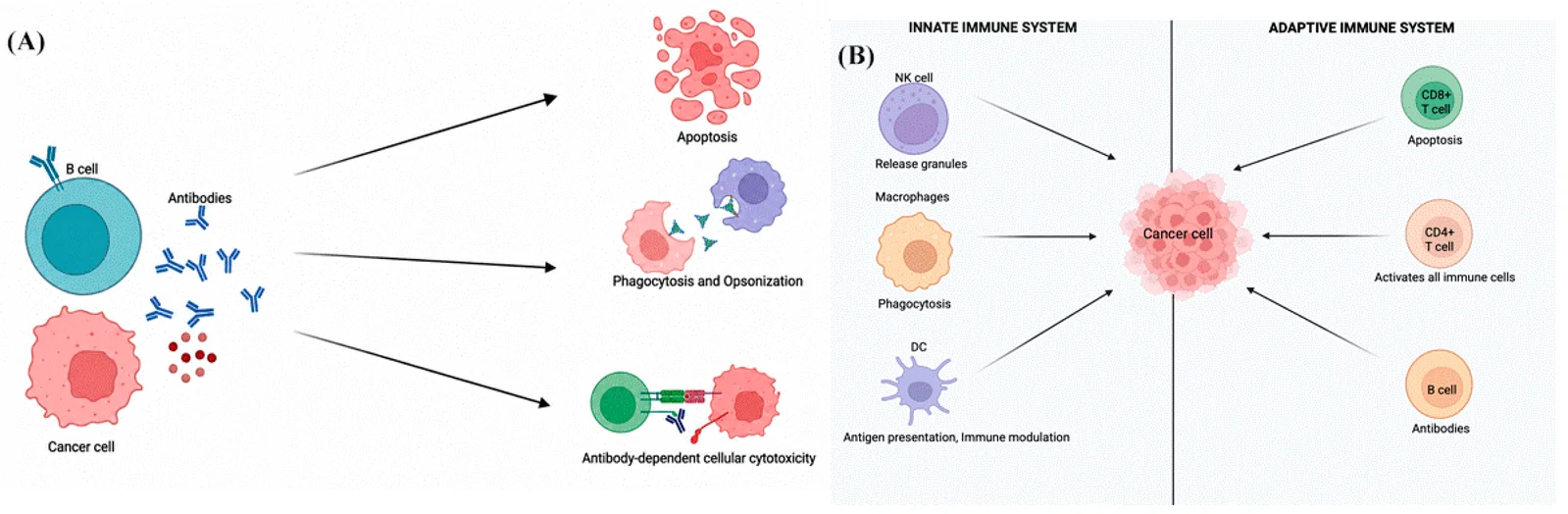
(**A**) B cell functionality in anti-tumor responses. B cells may mount an array of immune responses to tumor cells such as secretion of tumor-specific antibodies (Abs) that may mediate anti-tumor effects via apoptosis, phagocytosis, opsonization and Ab-dependent cell-mediated cytotoxicity (ADCC). Their functions are shaped by interactions with surrounding immune cells in the tumor microenvironment (TME). (**B**) Coordinated interaction between innate and adaptive arms of immune system in targeting cancer cells. Innate immunity may be triggered by cytotoxic granules released by natural killer (NK) cells and macrophage-mediated phagocytosis. Antigen presenting cell (APC) activity and immune modulation by dendritic cells (DCs) bridge innate and adaptive immunity. Apoptosis mediated by CD8^+^ T cells and CD4^+^ T cells providing helper signals orchestrate the adaptive immune response by activating other immune cells, such as CD8^+^ T cells and B cells. B cells contribute through Ag presentation and by producing Abs that target tumor antigens (Ags).

Although the role of T cells in anti-tumor immunity is well established [11,12], the contribution of B cells remains less explored [8,9]. B cell-derived Abs can promote tumor clearance by inducing apoptosis, enhancing phagocytosis and opsonization and mediating Ab-dependent cell-mediated cytotoxicity (ADCC) (Figure 1A). B cells can also regulate other immune cells through Ag presentation, co-stimulation and cytokine secretion, in addition to the effects of Abs and immune complexes [13]. B cell gene signatures have been associated with favorable outcomes in cancers such as ovarian and breast cancer [14]. However, B cells comprise diverse functional subsets that can exert both pro- and anti-tumor effects, with their balance potentially shaping tumor growth and progression.

B cell function reflects the intersection of lineage state, Ag specificity, tissue location, cytokine exposure and metabolic availability. Hypoxia, lactate, nutrient competition, adenosine and prostaglandin E2 (PGE2) can alter immune cell activation and trafficking, while B cells can reciprocally shape myeloid and lymphoid responses through cytokines, Abs and metabolites. B cell-derived γ-aminobutyric acid (GABA) provides a striking example of bidirectional metabolic–immune coupling [15]. This review provides an understanding of the TME and immune escape, focuses on B cell states, their metabolic conditioning, tumor-type heterogeneity and their relationship to therapeutic response and resistance. We use B cell immunometabolism as the central framework linking metabolic conditions in the TME to B cell phenotype, differentiation and function. Specifically, we examine how metabolic cues may shift the balance between regulatory, IgA-skewed or metabolically constrained B cell states and Ag-presenting, IgG-biased, plasma cell or TLS-associated states, and how these states may in turn influence interactions with T cells, myeloid cells and tumor cells. The central premise of this review is therefore to directly address the emerging concept that B cells are not merely passive participants in tumor immunity but metabolically conditioned regulators of the TME, with their effects strongly dependent on tumor type, spatial context and treatment setting.

## 2. Immune Defenses Against Cancer

Innate and adaptive immune cells cooperate to recognize and eliminate malignant cells (Figure 1B). Natural killer (NK) cells, macrophages and dendritic cells (DCs) provide early effector and Ag-presenting functions to reduce inflammation caused by the disease. These immune cells slow tumor growth by either killing malignant cells non-specifically or triggering an adaptive immune response. While adaptive T and B lymphocytes generate Ag-specific responses [16,17,18,19], CD8^+^ T cells can directly lyse tumor cells, CD4^+^ T cells provide helper functions and B cells contribute through Ag presentation, Ab production, cytokine secretion and organization of local immune niches.

Numerous studies have highlighted the role of genetic and epigenetic alterations in cancer [4]. These changes can modify cell surface proteins and contribute to the expression of tumor-associated Ags (TAA) [20]. TAA can be targeted by Abs, which may recruit and activate complement through the classical pathway, thereby contributing to complement-dependent cytotoxicity and other Fc-mediated effector mechanisms. However, cancer cells employ diverse stratagems to elude host immune defenses.

The metabolic state of the tumor determines the conditions under which these immune responses occur. Rapid tumor growth increases oxygen consumption and nutrient utilization while altered glycolysis and mitochondrial metabolism contribute to lactate accumulation, extracellular acidification and depletion of glucose and amino acids. Hypoxia-inducible pathways, adenosine generation and inflammatory lipid mediators further modify immune cell trafficking and effector functions [5,21].

## 3. Tumor Microenvironment (TME) and the Metabolic Conditioning of B Cells

**Figure 2:**
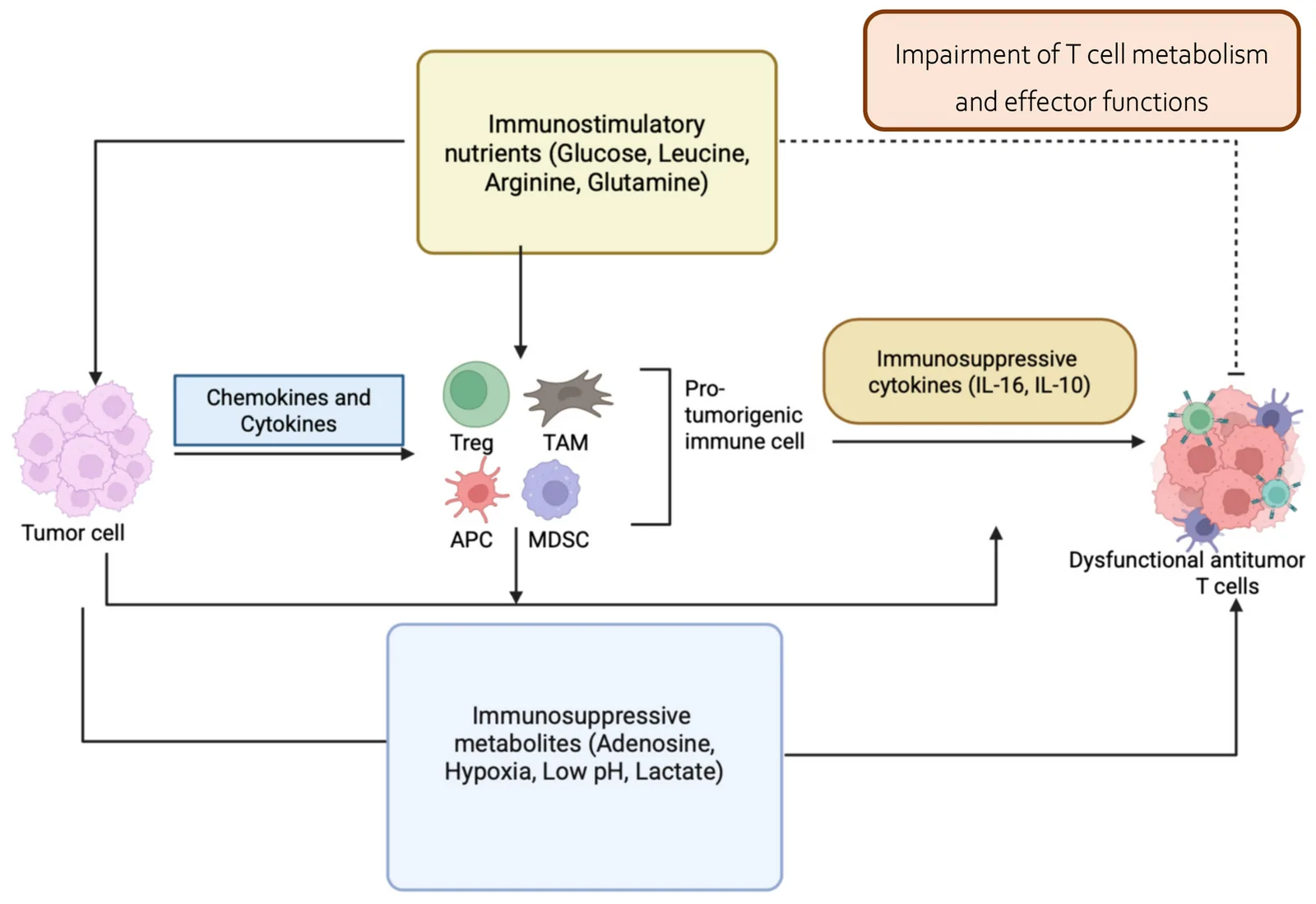
Immunosuppressive TME leading to T cell dysfunction in cancer. Chronic Ag stimulation coupled with metabolic stress within the TME promote T cell dysfunction and exhaustion. Immunosuppressive metabolites (adenosine, lactate), hypoxia, extracellular acidity and immunostimulatory nutrients (glucose, leucine, arginine, glutamine) can impair T cell metabolism and effector activity. Immunosuppressive populations, including regulatory T cells (Tregs), myeloid-derived suppressor cells (MDSCs) and tumor-associated macrophages (TAMs), further reinforce this dysfunctional state through inhibitory cytokines such as interleukin (IL)-10/IL-35 and metabolic signals. Together, these interconnected factors limit effective anti-tumor T cell responses and contribute to immune evasion.

The TME comprises malignant cells, fibroblasts, endothelial and vascular cells, adipocytes embedded in a modified extracellular matrix (ECM) and diverse innate and adaptive immune populations together with their secreted cytokines, chemokines and growth factors (Figure 2). Its composition varies substantially among tumor types and even among spatial regions of the same tumor [3,22,23,24]. Single-cell transcriptomic and spatial approaches have revealed that immune cell states are strongly associated with local nutrient availability, oxygenation, extracellular pH and cellular interactions. Chronic Ag stimulation along with exposure to inhibitory cytokines like interleukin (IL)-10, hypoxia, nutrient competition, lactate accumulation, extracellular acidity, immunosuppressive metabolites such as adenosine and immunostimulatory nutrients like glucose, leucine, arginine and glutamine can impair immune cell function and remodel the TME [5]. Although many of these effects are best established for T cells and myeloid cells, the same metabolic constraints may shape B cell activation, differentiation and function. This distinction is important because direct mechanistic evidence for human tumor-infiltrating B (TIL-B) cells remains comparatively limited. Metabolically distinct niches may therefore support different B cell differentiation states within the same tumor and metabolic stress may influence the balance between Ag-presenting/effector B cells and Breg populations. The potential consequences for therapy may consequently be interpreted as context-dependent rather than as evidence for a uniform B cell response to metabolic stress.

**Figure 3:**
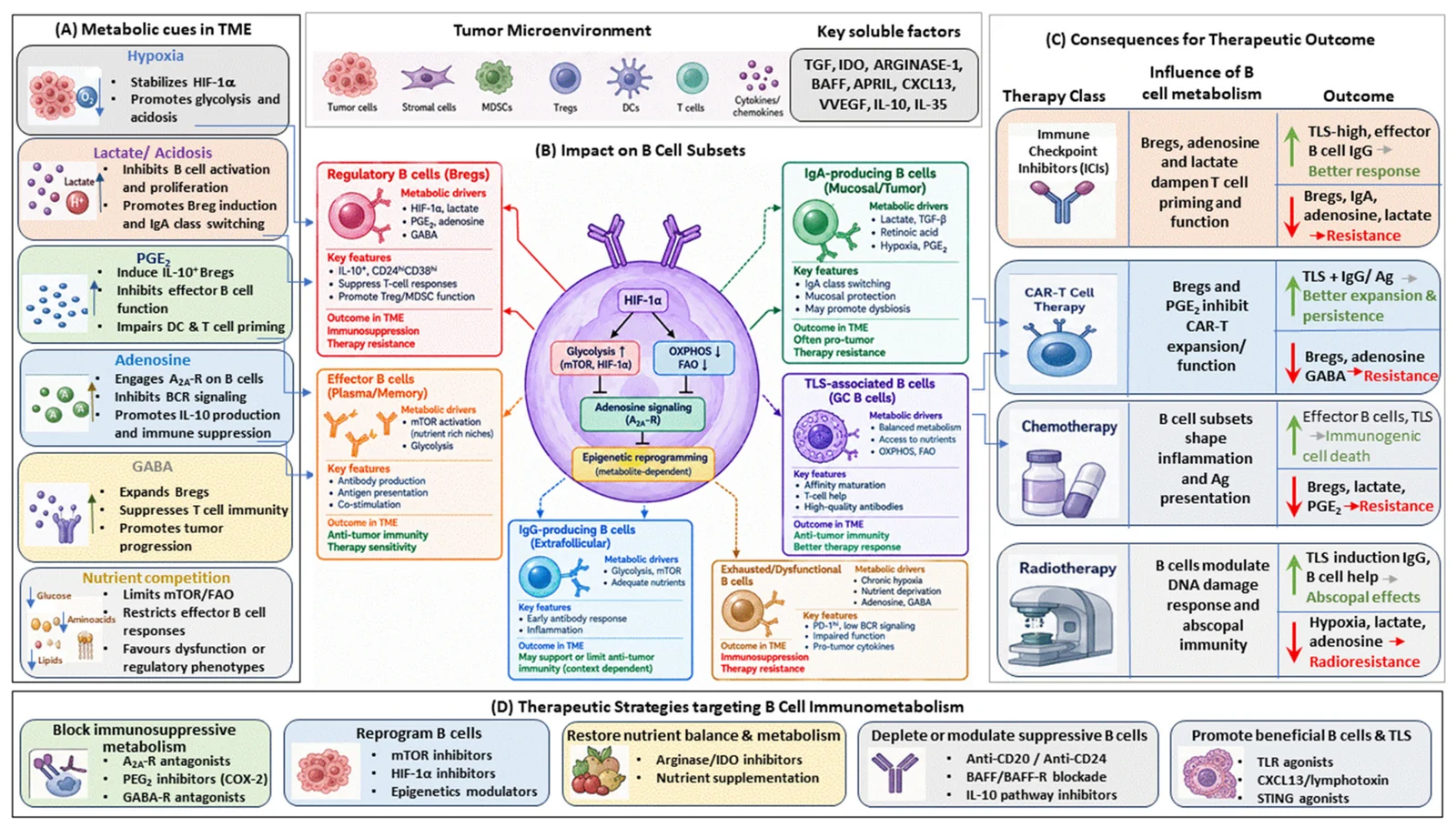
B cell immunometabolism in the TME. (**A**) Metabolic cues reprogram B cell states and thereby shape response to cancer therapies. The TME exposes B cells to hypoxia, lactate accumulation, extracellular acidosis, nutrient competition, adenosine, prostaglandin E2 (PGE2) and γ-aminobutyric acid (GABA), which may alter B cell activation, metabolism and differentiation. Although several of these metabolic effects are well established in other immune populations, their direct effects on human tumor-infiltrating B (TIL-B) cell subsets remain incompletely defined. While lactate buildup and acidosis can disrupt normal B cell activation and promote regulatory phenotypes, hypoxia can stabilize hypoxia-inducible factor 1-alpha (HIF-1α) and boost glycolytic activity. PGE2, adenosine and GABA can enhance immunoregulatory B cell responses and further restrict immune cell activity. Infiltrating B cells may have fewer metabolic resources due to competition with rapidly proliferating tumor cells for glucose, lipids, and amino acids. (**B**) The metabolic conditions may translate into different B cell states. Regulatory B cells (Bregs) associated with hypoxia, lactate, PGE2, adenosine and GABA may produce IL-10 and promote suppressive Treg and myeloid cell functions, potentially contributing to resistance to immune checkpoint blockade (ICB). In contrast, metabolically active effector, plasma cell, memory-like and TLS-associated B cells require active glycolysis and mechanistic target of rapamycin (mTOR) signaling to support Ab production, Ag presentation, co-stimulation and T cell responses, thereby contributing to anti-tumor immunity. Thus, Bregs associated with suppressive cytokines and metabolites may contribute to immune regulation, whereas metabolically competent effector, plasma cell, memory-like and TLS-associated B cells may support Ab production, Ag presentation, co-stimulation and T cell responses. Immunoglobulin (Ig)A- and IgG-producing B cells can have context-dependent effects. IgA- and IgG-producing B cells may have context-dependent effects. (**C**) Potential consequences for therapeutic outcome. While Bregs and associated suppressive networks/metabolically restrictive factors may lead to resistance to immune checkpoint inhibitors (ICI), effector and TLS-associated B cell responses may promote higher anti-tumor immunity. Similarly, for chimeric antigen receptor T (CAR-T) cell therapy, chemotherapy and radiotherapy, B cell effects are currently less well defined and may occur indirectly through broader changes in the TME and immune cell function. For CAR-T cell therapy, regulatory elements including PGE2, adenosine and GABA may impede T cell activity while efficient B cell Ag presentation and supporting immunological organization may promote CAR-T cell expansion and durability. B cell populations can affect Ag presentation, inflammation and the effects of immunogenic tumor cell death during chemotherapy. Additionally, radiation therapy can modify TLS and B cell responses, which may contribute to immune-mediated effects other than direct tumor cell DNA damage. These relationships may therefore be regarded as emerging hypotheses rather than established B cell-specific resistance mechanisms. (**D**) Potential therapeutic strategies to alter B cell immunometabolism. These include modulation of mTOR/HIF-1α signaling, restoration of metabolic resources, inhibition of suppressive metabolic pathways and selective targeting of regulatory B cell programs while preserving or enhancing beneficial B cell and TLS responses. These approaches may influence responses to immune checkpoint blockade (ICB), CAR-T cell therapy, chemotherapy and radiotherapy. Overall, the therapeutic goal is not broad B cell depletion, but selective metabolic and functional reprogramming toward B cell states to boost durable anti-tumor immunity. Abbreviations: BAFF, B cell activating factor; APRIL, a proliferation-inducing ligand; CXCL13, C-X-C motif chemokine ligand 13; VEGF, vascular endothelial growth factor; IDO, indoleamine 2,3-dioxygenase; ARG1, arginase 1; A2A-R, adenosine A2A receptor; OXPHOS, oxidative phosphorylation; FAO, fatty-acid oxidation; COX-2, cyclooxygenase-2; TLR, Toll-like receptor; STING, stimulator of interferon genes.

Recent studies have linked the TME composition to cancer prognosis and treatment outcomes [3]. The dynamic interplay between cancer cells and TME components begins early in tumor development and sustains tumor survival, local invasion, metastasis and angiogenesis and may be prospective targets for therapy [22]. The TME also adapts to hypoxic and acidic conditions by promoting angiogenesis to replenish the oxygen and nutrient supply and eliminate metabolic waste. Both innate and adaptive immune cells infiltrate the tumors and can either promote or restrain tumor progression [23]. For instance, renal cell carcinomas (RCC) may enhance the recruitment and activation of non-tumor stromal and immune cells through the release of growth factors and cytokines and modify the TME via hypoxia and necrosis [24]. The TME shapes B cell phenotype and function through hypoxia, nutrient competition, lactate accumulation, extracellular acidity, adenosine, PGE2 and suppressive cytokine networks. These signals can influence B cell activation, differentiation, Ab production and regulatory functions, while also affecting their interactions with T cells, myeloid cells and other components of the tumor ecosystem. The varied metabolic stress or cues and their impact on B cell functional states within the TME that drive the therapeutic consequences are depicted in Figure 3 and Table 1.

### 3.1. Hypoxia and Nutrient Competition

Oxygen limitation can alter cellular signaling and transcriptional programs within immune cells and may favor immunosuppressive interactions in the TME. Tumor hypoxia is associated with hypoxia-inducible factor (HIF)-dependent metabolic adaptation, abnormal vasculature and suppression of immune effector functions. Hypoxia may alter activation, differentiation, trafficking and Ab responses in B cells indirectly through changes in stromal and myeloid cell signals [5]. However, the direct metabolic consequences in human TIL-B subsets remain incompletely defined. Glucose, glutamine, arginine and other nutrients are simultaneously consumed by tumor cells and competing immune populations, creating a resource-limited environment. Therefore, the presence of B cells cannot be interpreted merely as evidence of effective humoral immunity. Rather, their functional competence may depend on access to metabolic substrates and supportive niches.

### 3.2. Lactate and Acidity

Increased glycolytic flux in tumors leads to extracellular lactate accumulation and acidification that can impair immune cell signaling and promote immunosuppressive myeloid programs [5,21]. Although the B cell-specific literature remains less developed than that for T cells and macrophages, lactate-rich niches are likely to influence B cell activation and interaction with antigen-presenting cells (APCs) and myeloid cells.

### 3.3. Adenosine and PGE2

Adenosine generated through extracellular nucleotide metabolism such as adenosine triphosphate (ATP) catabolism and lipid-derived mediators like PGE2 produced through cyclooxygenase pathways are prominent immunoregulatory signals in many tumors that can contribute to an immunosuppressive milieu favorable for tumor persistence, alter APC function, immune cell recruitment and promote myeloid cell accumulation [25]. Their effects on B cells may be subset-specific, influencing activation, cytokine production and differentiation. Though the effects of these metabolites on T cells and myeloid cells have been well established, direct evidence connecting these pathways to B cell-mediated treatment resistance remains limited.

### 3.4. Kynurenine and Amino Acid Metabolism

Indoleamine 2,3-dioxygenase (IDO) and tryptophan 2,3-dioxygenase (TDO)-mediated tryptophan catabolism generates kynurenine and contributes to immune suppression. Amino acid deprivation and altered arginine metabolism can similarly constrain immune cell function [26]. Whether these metabolic pathways directly select for Breg, IgA-producing or TLS-associated B cell states in human tumors is not yet fully understood. Nevertheless, they represent plausible mechanisms linking the metabolic architecture of the TME to B cell functional plasticity.

Thus, metabolic stress does not act as a single binary suppressive switch. Instead, it can redistribute B cell states and alter their communication with T cells, myeloid cells, stromal cells and tumor cells. This provides a mechanistic framework for understanding why B cell abundance has divergent prognostic associations across cancers. Tumor immunometabolism is an important determinant of B cell functional plasticity rather than an independent feature of the TME [27]. Understanding how metabolic signals selectively shape regulatory versus effector B cell states may provide opportunities to preserve beneficial B cell functions while limiting immunosuppressive activities.

## 4. Tumor Immune-Evasion Stratagems and Their Metabolic Dimensions

**Figure 4:**
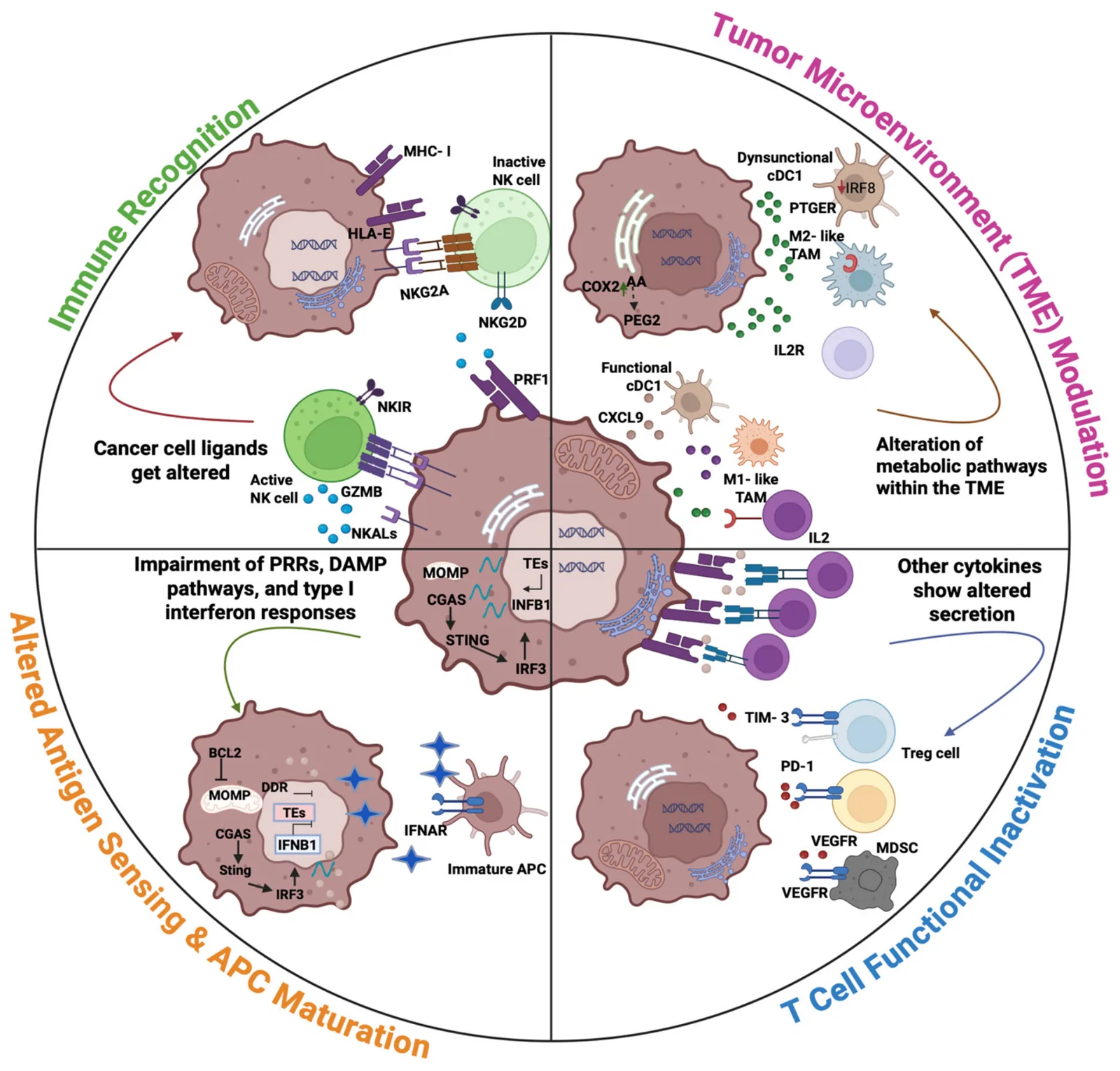
Tumor immune-evasion strategies and metabolic reinforcement. Tumors evade immune attack by reducing Ag presentation, remodeling immune cell recruitment, increasing immunosuppressive mediators and disrupting innate immune sensing. They impair NK cell activation by reducing NK cell activating ligands (NKALs) while increasing inhibitory signals and NK cell inhibitory ligands (NKILs) and promote immunosuppressive CD4^+^CD25^+^FOXP3^+^ Treg, MDSC and TAM activity. Tumors suppress innate immune sensing by disrupting pattern recognition receptor (PRR), damage-associated molecular pattern (DAMP), and type I interferon (IFN) signaling pathways. Tumors remodel their secretome by upregulating immunosuppressive factors (VEGFA), which signal through VEGFR (kinase insert domain receptor, KDR) to impair effective immune cell recruitment within the TME. Metabolic reprogramming reinforces these suppressive mechanisms and promotes immune escape through COX-2/prostaglandin-endoperoxide synthase 2 (PTGS2)-driven PGE2 production and lactate-associated signaling. PGE2 suppresses APCs, DCs, NK cells and cytotoxic T lymphocytes (CTLs) while promoting MDSCs and TAM functions. Collectively, tumors impair T cell receptor (TCR) and IL-2 signaling, cytotoxic effector functions, immune checkpoint regulation, DNA damage responses and cellular stress pathways, ultimately driving T cell exhaustion and tumor immune escape.

The widely accepted ‘three Cs’ concept proposes that the host immune system eliminates emerging malignant cell precursors and maintains microscopic neoplasms in dynamic equilibrium, preventing cancer from metastasizing until neoplastic cells develop genetic or epigenetic changes that enable immune escape. Tumor cells evade immune surveillance through the ‘three Cs’ conceptual framework or key strategies: (1) camouflage, which limits immune recognition; (2) coercion, which suppresses or interferes with immune effector functions; and (3) cytoprotection, which shields malignant cells from immune cytotoxicity [23]. Targeting these mechanisms may enhance the efficacy of immunotherapy and conventional anticancer treatments. Tumors evade immune destruction through impaired Ag presentation, immunosuppressive soluble mediators, checkpoint signaling and recruitment or reprogramming of suppressive immune populations [25,28,29,30,31,32]. The diverse immune-evasion strategies adopted by neoplastic cells are summarized in Figure 4.

### 4.1. Downregulation of Ag Presentation

Cancer cells evade immune recognition by reducing major histocompatibility complex (MHC)-I and Ag processing machinery such as transporters associated with peptide processing (TAP)1/2 and nucleotide-binding domain (NLR) family caspase recruitment domain (CARD) domain containing 5 (NLRC5), through genetic and epigenetic alterations, thereby impairing CD8^+^ T cell recognition and promoting immune escape and immunotherapy resistance [24,29].

### 4.2. Secretion of Immunosuppressive Factors

Tumors and associated stromal/immune cells release factors such as tumor growth factor (TGF)-β, IL-10, vascular endothelial growth factor (VEGF), PGE2, IDO-derived metabolites and adenosine that dampen immune cell function and trafficking to tumors, deplete essential metabolic nutrients for the immune cells, establish a tolerogenic TME and facilitate tumor progression and immune evasion [25].

### 4.3. Immune Checkpoint Activation

Cancer cells exploit inhibitory checkpoints such as programmed cell death protein (PD)-1/programmed cell death ligand (PD-L)1, cytotoxic T lymphocyte-associated protein (CTLA)-4, lymphocyte-activation gene (LAG)-3 and T cell immunoglobulin and mucin-domain (TIM-3) to restrain T cell activation and promote T cell exhaustion, thereby weakening anti-tumor immunity [30].

### 4.4. Recruitment of Immunosuppressive Cells

Tumor-derived chemokines such as chemokine C-C motif ligand 2 (CCL2), C-X-C motif chemokine ligand (CXCL)12, CXCL8 and metabolic signals recruit immunosuppressive cells such as Tregs, TAMs and MDSCs to the TME, where they suppress anti-tumor immune responses, promote angiogenesis and tumor progression and contribute to metastasis and therapeutic resistance [31,32].

The immune escape mechanisms are interconnected with tumor metabolism rather than operating independently. Hypoxia and lactate can promote suppressive myeloid phenotypes, adenosine can restrain immune activation, PGE2 can alter Ag-presenting and effector cell functions and nutrient competition can restrict the capacity of immune cells to sustain cytotoxic functions. Lactate production further activates hydroxycarboxylic acid receptor (HCAR)1, thereby promoting the recruitment of polymorphonuclear (PMN)-MDSCs and secretion of inhibitory molecules. The recruited TAMs create an anti-inflammatory (M2-polarized) environment through cytokines (IL-10 and IL-35), promoting angiogenesis and reprograming towards immunosuppression [33]. The metabolic cues in the TME that shape the phenotype and function of distinct B cell subsets resulting in divergent outcomes on response or resistance to therapy are depicted in Figure 3.

Immune evasion is particularly relevant for B cells because the same TME signals that suppress cellular immunity can alter B cell differentiation. Tumor-associated Bregs can reinforce suppression through IL-10 or IL-35, while B cell-derived GABA can promote IL-10 producing macrophages [15]. Conversely, TLS-associated B cells may sustain Ag presentation, clonal expansion and Ab responses in spatially organized niches. Thus, tumor immune escape is a consequence of competition between metabolically conditioned immune states rather than due to uniform suppression of B cells.

## 5. Role of T Cells in Cancer and Their Interaction with B Cells

**Figure 5:**
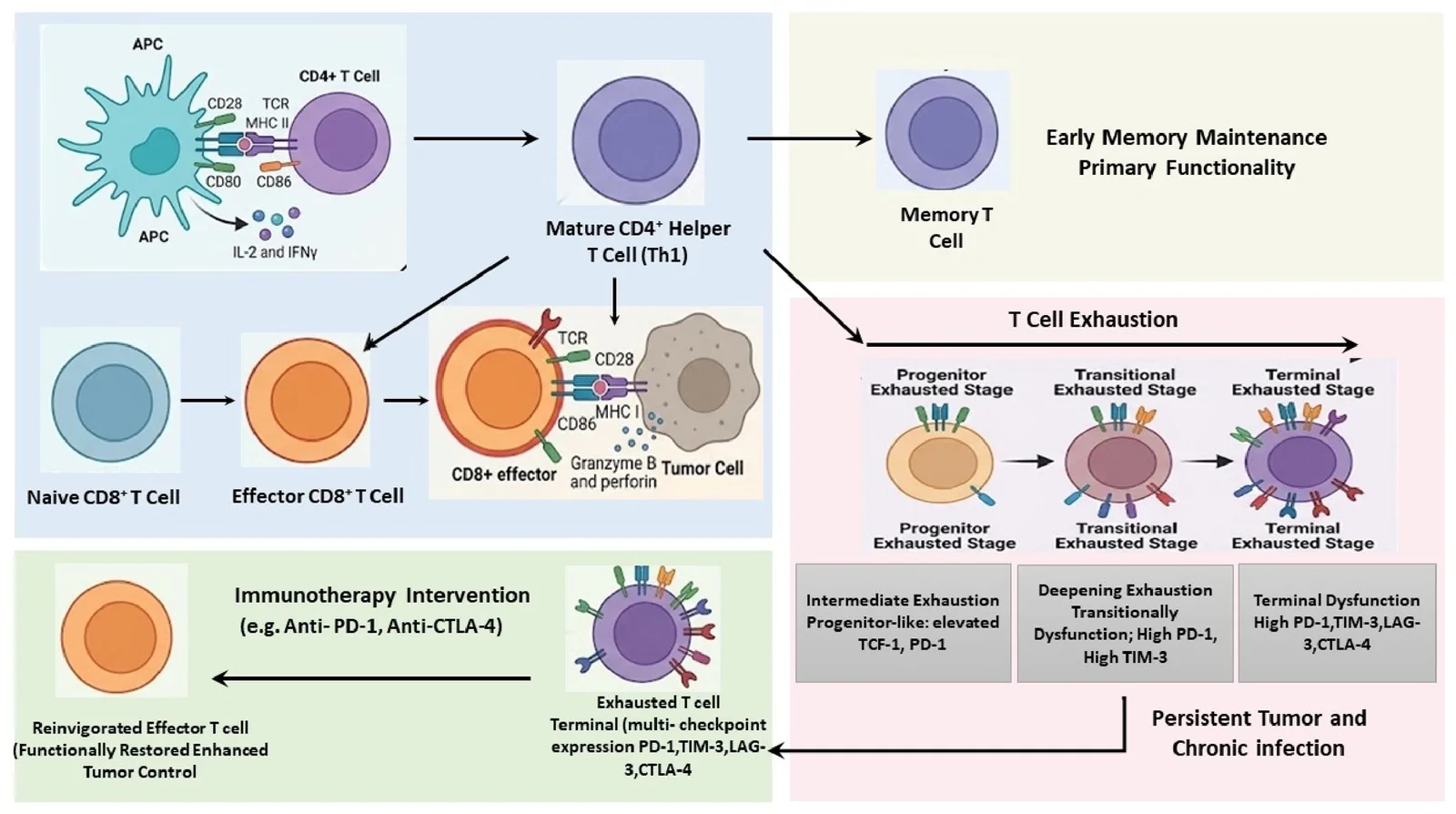
T cell exhaustion in cancer and its context for B cell-mediated immune regulation. Persistent Ag exposure and metabolic stress within the TME drive progressive T cell dysfunction and exhaustion. Ag presentation and CD80/CD86-CD28 co-stimulation activates CD4^+^ T cells, while IL-2 and IFN-γ promote Th1 differentiation and CD8^+^ T cell-mediated tumor killing through perforin and granzyme B. Chronic stimulation due to persistent Ag exposure within TME drives T cells through a continuum of exhaustion, progressing from a progenitor exhausted state (T cell factor {TCF}-1^high^, programmed cell death protein {PD}-1^intermediate^) to a transitional exhausted state (PD-1^high^, T cell immunoglobulin and mucin-domain {TIM}-3^high^) and ultimately to a terminal exhausted state characterized by profound functional impairment and sustained expression of multiple inhibitory receptors (PD-1, TIM-3, lymphocyte-activation gene {LAG}-3 and cytotoxic T lymphocyte-associated protein {CTLA}-4). B cell Ag presentation, cytokine production and TLS organization may either support anti-tumor T cell responses or contribute to immune regulation. Immune checkpoint blockade, particularly PD-1 and CTLA-4 inhibition, can partially restore T cell function and reinvigorate anti-tumor activity.

T lymphocytes are central mediators of adaptive anti-tumor immunity and provide an important context for understanding the complementary and opposing functions of B cells. CD8^+^ cytotoxic T lymphocytes recognize TAA presented by MHC-I and can eliminate malignant cells through perforin- and granzyme-mediated cytotoxicity, whereas CD4^+^ T cells regulate anti-tumor responses through cytokine production and interactions with other immune populations [34,35,36,37,38,39]. However, persistent Ag exposure within the TME, together with hypoxia, nutrient limitation and other immunosuppressive conditions, can progressively impair T cell effector function and promote an exhausted state characterized by increased expression of inhibitory checkpoints such as PD-1, TIM-3, CTLA-4 and LAG-3 [40,41,42,43]. The T cell exhaustion events in cancer is depicted in Figure 5.

The reciprocal interaction between T and B cells is particularly important within TLSs. B cells can present TAA through MHC-II, provide co-stimulatory signals and produce cytokines that influence T cell activation, while T cell-derived signals regulate B cell activation, GC responses, class switching and Ab production. These interactions can support sustained anti-tumor immunity when they occur within an organized and metabolically permissive TME. Conversely, metabolic stress including hypoxia, glucose and nutrient competition, lactate accumulation, extracellular acidity and adenosine, can impair T cell function while simultaneously altering B cell activation and differentiation [44]. Thus, the outcome of T-B cell interactions is determined not only by Ag recognition and cytokine signaling but also by the metabolic context in which these cells operate, providing an important link between B cell immunometabolism and therapeutic response or resistance.

## 6. B Cell States in the Metabolically Altered TME

**Figure 6:**
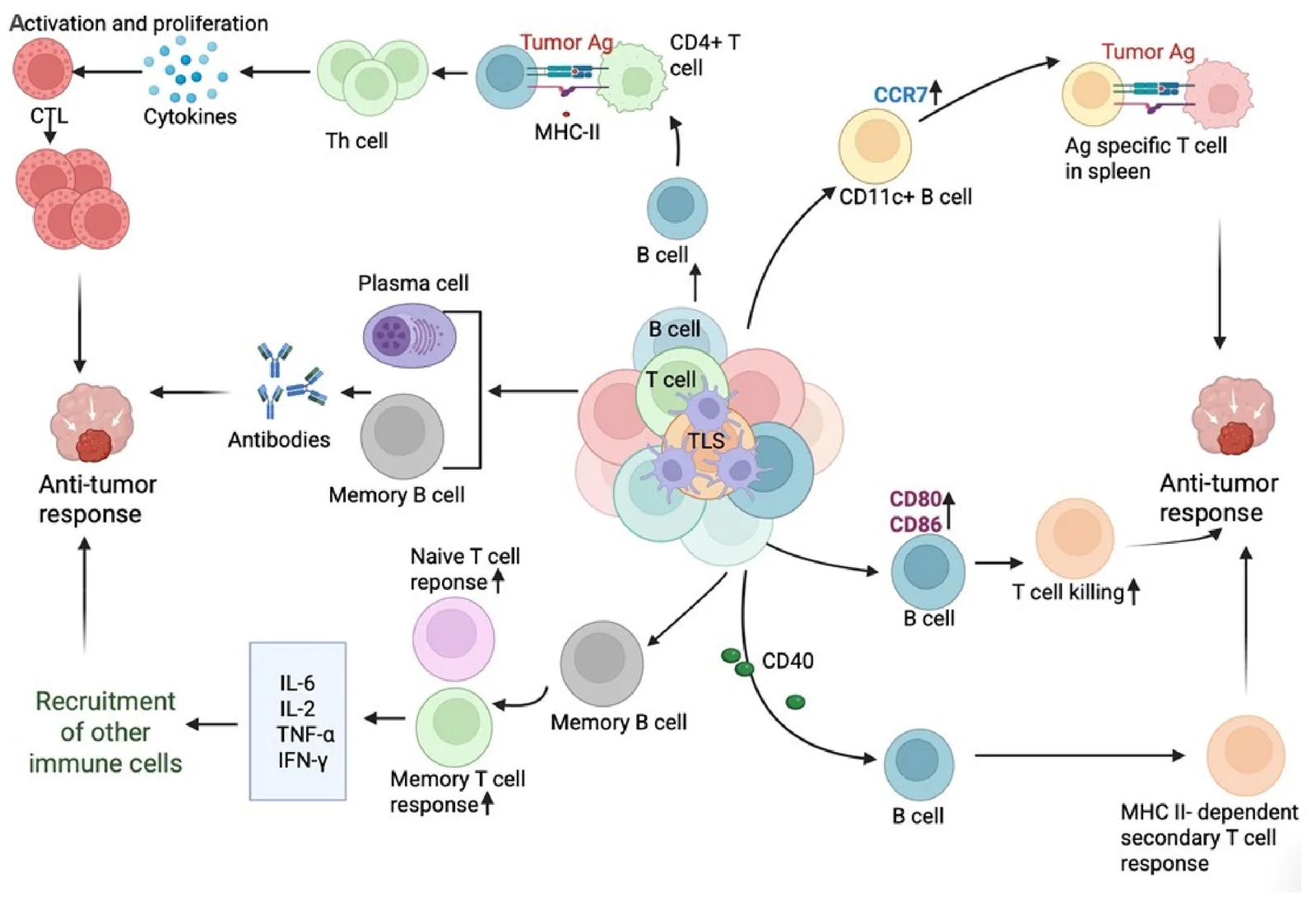
Role of TLSs and B cells in anti-tumor immunity. TLSs serve as specialized immune niches where B cells, T cells and APCs orchestrate adaptive anti-tumor responses. TLS-associated B cells process and present tumor Ags to CD4^+^ Th cells, leading to cytokine production that promotes the activation, proliferation and cytotoxic function of CD8^+^ T cells. Activated B cells undergo clonal expansion, affinity maturation, somatic hypermutation and class-switch recombination, differentiating into memory B cells and plasma cells that produce tumor-specific Abs. A distinct population of CD11c^+^CCR7^+^ B cells, located at the T–B cell interface, exhibits potent APC capacity and efficiently activates Ag-specific T cells. Besides Ab production, TIL-Bs provide essential co-stimulatory signals through CD80 and CD86 and secrete cytokines (TNF-α, IL-2, IL-6 and IFN-γ) which enhance T cell activation and recruit additional immune cells into the TME and help sustain local anti-tumor immunity. TLS-associated B cells play pivotal roles in Ag presentation, humoral immunity and orchestration of durable anti-tumor responses, highlighting their potential as therapeutic targets in cancer immunotherapy. The function of TLS-associated B cells is likely influenced by local metabolic conditions, including oxygenation, nutrient availability and metabolite gradients.

The immune response orchestrated within TLS with T-B and APC interaction is depicted in Figure 6. While B cells differentiate into memory B cells and Ab-producing plasma cells that target tumor cells directly, they also present TAA via MHC-II molecules to CD4^+^ T helper (Th) cells, which in turn secrete cytokines to activate cytotoxic T lymphocytes (CTLs), enhancing their proliferation and killing tumor cells. B cells provide essential co-stimulatory signals (CD80/CD86) to boost T cell-mediated tumor destruction. A subpopulation of B cells (CD11c^+^ B cells) exhibiting high levels of C-C chemokine receptor type 7 (CCR7) at the interface of splenic T and B cell zones effectively present Ags, activating T cells. TIL-Bs and their Abs mediate immune response against cancer, release a mix of pro- and anti-inflammatory cytokines and produce tumor-specific Abs. Memory B cells support both memory and naïve T cell responses in tumors and secrete cytokines such as tumor necrosis factor (TNF)-α, IL-2, IL-6 and interferon (IFN)-γ, which help recruit and activate additional immune cells [45]. TIL-Bs thus play a crucial role in shaping anticancer immune response, offering promising targets for the development of future immunotherapies.

Thus, B cells in cancer comprise a continuum rather than a single population. Their phenotype can range from regulatory and metabolically suppressive states to Ag-experienced effector, plasma cell and TLS-associated states. The balance is influenced by BCR signaling, TLR stimulation, cytokines, Ag load, tissue location and metabolic conditions. The B cell states may be viewed along a functional spectrum from Bregs and other suppressive populations at one end, to metabolically stressed, IgA-skewed or GABA-producing states in intermediate niches and Ag-presenting, clonally expanded, IgG-biased and TLS-associated B cells in immune-active niches.

### 6.1. Tumor-Promoting Roles of B Lymphocytes in the TME

**Figure 7:**
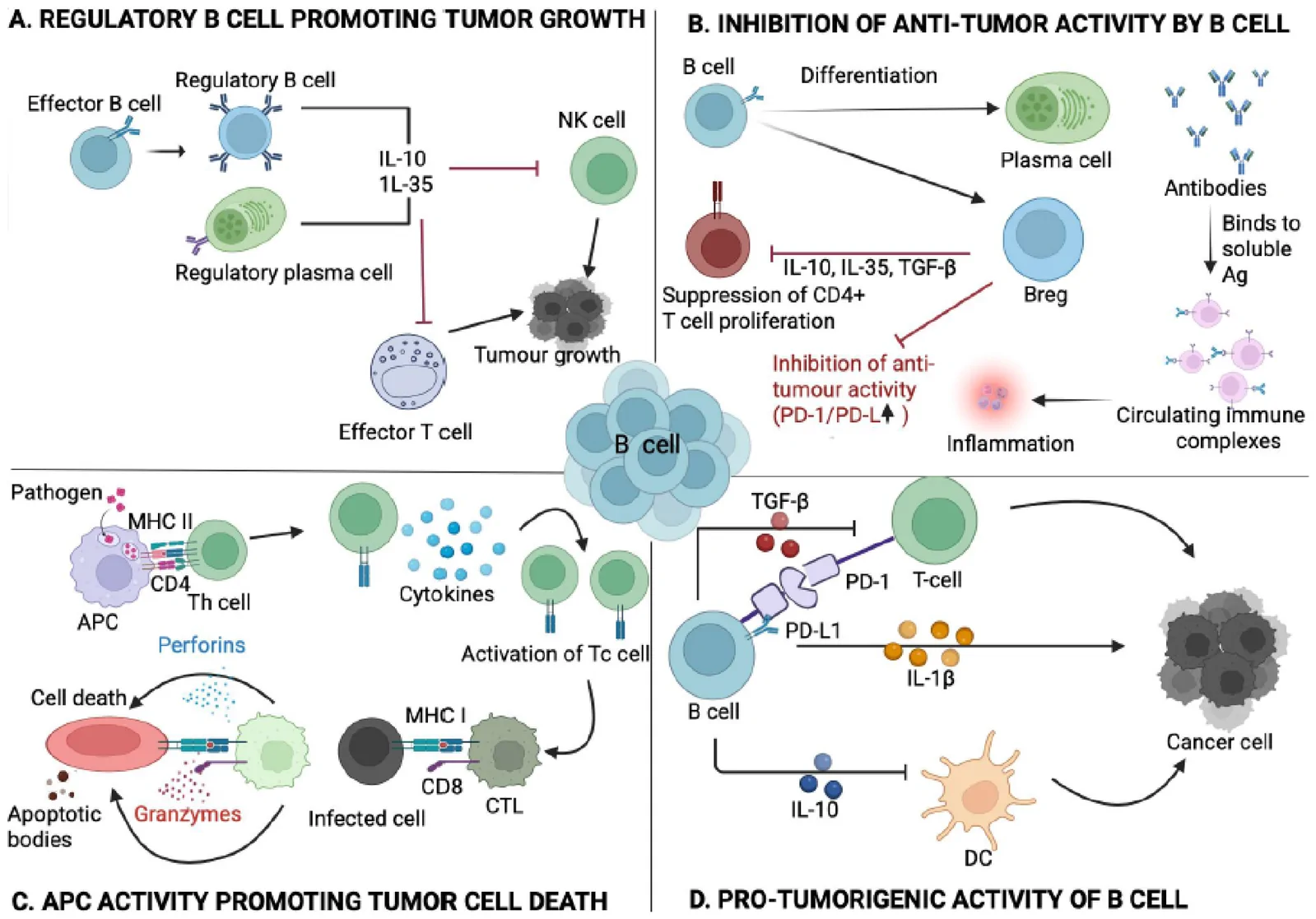
Dual role and immunometabolic spectrum of B cells in tumor immunity. B cells can either promote anti-tumor immunity or support tumor progression, depending on their functional state and the metabolic conditions of the TME. (**A**) Regulatory B cells and plasma cells release IL-10 and IL-35, suppressing effector T and NK cell activity and promoting T cell exhaustion and tumor growth. (**B**) B cells may inhibit anti-tumor responses through IL-10, IL-35, TGF-β and PD-1/programmed cell death ligand (PD-L)1 signaling. Abs binding soluble tumor Ags can form circulating immune complexes that promote inflammation and tumor-supportive activity. (**C**) Ag-presenting B cells activate CD4^+^ T cells through MHC class II and co-stimulatory signals, thereby supporting cytotoxic T lymphocyte (CTL) responses and tumor cell killing. B cells may also contribute directly to tumor cell death through perforin, granzymes, Fas ligand and Ab-dependent mechanisms. (**D**) B cells can promote immune suppression through TGF-β, IL-10, PD-1/PD-L1 signaling and B cell-derived GABA, which may impair DC and T cell function and support tumor progression. Metabolic cues including hypoxia, lactate, nutrient competition, adenosine, PGE2 and kynurenine may further shape these distinct B cell states and influence responses to cancer therapy such as checkpoint blockade, chemotherapy, radiotherapy and cellular therapies.

The complex roles played by B cells in tumor progression and immune regulation are discussed below and depicted in Figure 7. B cells exert both anti-tumor and pro-tumor effects within the TME. While Ag-presenting and TLS-associated B cells promote CD4^+^ and CD8^+^ T cell responses and Ab-mediated tumor control, Bregs and regulatory plasma cells suppress anti-tumor immunity through IL-10, IL-35, PD-1/PD-L1 signaling, limiting T- and NK cell activity, impairing DC function and promoting T cell exhaustion, thereby creating a tumor-supportive microenvironment that facilitates immune evasion and tumor progression. Metabolic cues including hypoxia, lactate, nutrient competition, adenosine, PGE2, kynurenine and B cell-derived GABA may shape these distinct B cell states and influence responses to checkpoint blockade, chemotherapy, radiotherapy and cellular therapies.

#### 6.1.1. Regulatory B Cells (Bregs) and Metabolically Supported Immune Suppression

Bregs are functionally defined by their capacity to suppress inflammation rather than by a single universally accepted phenotype. IL-10-producing Bregs have been described in cancer and may preferentially express markers such as CD9 or TIM-1 [46,47]. Bregs may potentially encourage the growth of tumors via the secretion of IgG4 Abs, angiokine synthesis or processes involving the dampening of anti-tumor immune responses [48]. In pancreatic cancer models, CD1d^hi^CD5^+^ B cell populations have been associated with tumor-promoting activity, with IL-35, rather than IL-10, implicated in murine Kras-driven pancreatic neoplasms, human pancreatic intraepithelial neoplasia and ductal adenocarcinoma lesions, mediating pro-tumorigenic effect (Figure 7A). Adoptive transfer of CD1d^hi^CD5^+^ B cells have been reported to restore tumor growth, and B cell-deficient mice display fewer pancreatic neoplasms coupled with increased T and NK cell responses [49]. Adoptive transfer of wild-type (WT) mice with IL-10^−/−^ B cells could restore tumor development, suggesting that IL-10 is not necessary for tumor growth inhibition [50].

In a mouse model of melanoma, selective deficiency of phosphatase and TENsin homolog deleted on chromosome 10 (PTEN) in B cells resulted in increased tumor development and reduced IFN-γ- and TNF-α-producing CD8^+^ T cells. Additionally, these mice displayed higher CD19^+^CD5^+^CD43^+^ B1a Breg tumor invasion. Melanoma growth was accelerated by adoptively transferring these B1a cells from WT mice, but not from IL-10^−/−^ mice [51]. These results suggest that B1a-like Breg cells promote melanoma growth through IL-10 in murine models and could be a possible therapeutic target [51]. Further studies are needed to validate these findings in humans, particularly given the ongoing debate over the existence of human B1a cells and the specificity of CD5 as a defining marker.

Metabolically, Breg function may be considered within the broader suppressive network of hypoxia, adenosine, lactate and lipid mediators. These signals may favor cellular programs that limit inflammatory effector responses, although direct Breg-specific causal data are still lacking. Metabolic stress can create a permissive niche for Breg activity through coordinated effects on B cells and neighboring myeloid/stromal cells. Future studies may determine whether distinct metabolic signatures can prospectively identify suppressive B cell states in human tumors.

#### 6.1.2. Antigen (Ag)-Presenting B Cells in Immune Activation-Context-Dependent Outcomes

B cells laden with antigenic peptides can function as professional APCs and interact with both CD4^+^ and CD8^+^ T cells, activating them and triggering Th1- and Th2-type downstream adaptive immune responses [52]. B cells also cross-present in the secondary lymphoid organs. Ags displayed on the surface of resident DCs or those near high endothelial venules (HEV) in the paracortex may be encountered by B cells migrating via the lymph nodes. In tumors, however, the consequence of B cell Ag presentation depends on its activation state, Ag specificity and local signals [53,54,55,56].

A metabolically permissive, inflammatory niche may support Ag presentation and co-stimulation, whereas hypoxia, nutrient limitation and suppressive mediators may constrain B cell activation or redirect it towards regulatory functions (Figure 7). Thus, B cell APC activity may or may not be considered intrinsically anti-tumor, depending on the B cell state presenting Ag, the metabolic conditions and the co-stimulatory and cytokine context.

#### 6.1.3. Dual Immunoregulatory Roles of B Cells in Tumorigenesis-B Cell-Derived GABA as a Metabolic Immune Circuit

In addition to the cytokines and Abs, B cells also release a few additional substances that influence tumorigenesis (Figure 7D). The development of lymphoid organs, particularly ectopic tertiary lymphoid organs (TLOs), depends critically on lymphotoxin α1β2 (LTα1β2) [57]. B cell-derived GABA illustrates a direct link between B cell metabolism and immune regulation. In a murine MC38 colon cancer model, B cell-derived GABA promoted IL-10-producing macrophages and limited anti-tumor immunity [15], while selective depletion of B cell-derived GABA restored anti-tumor responses [58]. B lymphocytes in TLOs have also been reported to be linked to enhanced anti-tumor immunity in the lungs [59]. The study provides strong mechanistic evidence in mice, but the extent to which an analogous GABA-producing B cell program operates in human solid tumors remains to be established. Accordingly, GABA should be regarded as a compelling candidate pathway rather than a clinically validated universal mechanism of B cell-mediated resistance.

#### 6.1.4. Antibody (Ab)-Producing Effector B Cell Subsets Having Pro-Angiogenic and Tumor-Supporting Role

Abs can also promote tumor progression [60]. By binding soluble tumor Ags, they form circulating immune complexes (CICs) that are often associated with poor clinical outcomes [60,61] (Figure 7B). These immune complexes can activate FcγR-bearing myeloid cells, promote inflammatory and pro-angiogenic responses, and recruit tumor-supportive cells such as macrophages and mast cells, thereby facilitating tumor persistence and vascularization [61,62].

In addition to IgG, IgA’s role in tumor immunity adds another level of intricacy [63]. Although there is ongoing discussion on their exact function in tumor growth, recent research has demonstrated that IgA-producing B cells frequently infiltrate and are located within the TME [64]. In contrast to IgG, immunosuppressive signals frequently drive IgA synthesis in some tumors, where IgA-dominated humoral responses have been associated with unfavorable pathogenic outcome [65,66,67]. The functional consequence may depend on Ag specificity, tissue compartment, Fc receptor engagement, the inflammatory environment and the signals driving class switching. IgA maintains an anti-inflammatory environment in mucosal tissues, which helps to promote immunological tolerance. Interestingly, elevated inflammatory responses have been associated with IgA deficit, indicating a homeostasis function [68]. By causing monocytes to secrete IL-10 and maybe through interactions with MZB1, a protein unique to marginal zone B and B1 cells, IgA may further reduce anti-tumor immunity by enhancing immunoregulatory signaling that supports tumor immune evasion [68].

### 6.2. Tumor-Suppressive Roles of B Lymphocytes in Anti-Tumor Immunity

#### 6.2.1. Direct Cytotoxic Activity of B Cells

Certain B cell populations have been reported to exert direct cytotoxic effects on tumor cell death-inducing Fas ligand (FasL)-mediated killing has been demonstrated principally in murine experimental systems [69,70] (Figure 7C), TIL-Bs can also directly induce tumor cell death through Ab-dependent mechanisms. Notably, anti-death receptor (DR)5 therapy loses efficacy in B cell-deficient animals such as colon adenocarcinomas and mammary murine cancer models, highlighting a key role for B cells in Ab-mediated tumor apoptosis [60]. Granzyme B, which has been shown to increase direct cytotoxicity against tumor cells in mouse models [71], has been reported to be secreted by IL-21-stimulated human B cells [72].

B cells can also produce chemokines and cytokines that influence immune cell recruitment. CCL22 can interact with CCR4 and affect T cell and regulatory cell trafficking, whereas CCL17 has context-dependent effects that may be anti-tumor or immunoregulatory [73,74,75,76,77]. The direction of these effects is determined by the cellular source, tumor type and local cytokine/metabolic environment.

#### 6.2.2. Immunomodulatory Cytokine Production by B Cells

Beyond Ab secretion, specifically, B cells produce cytokines, chemokines and other signaling molecules that regulate T cells, DCs, macrophages and NK cells and can promote anti-tumor immune cell recruitment and activation [73,74,75,76,77,78,79,80]. For instance, naïve B cells are known to produce chemokine ligands, CCL22 and CCL17 [73,74]. CCL22, predominantly secreted by activated B cells and DCs, interacts with CCR4 receptor to promote the migration of activated T cells [75]. Additionally, EBV-infected B cells have been shown to upregulate the expression of CCL17 and CCL22, which attract Tregs and Th2 cells, key players in immune modulation [76]. B cell-derived CCL3, CCL4 and CCL5 can attract and recruit Th1 and cytotoxic T cells to the tumor site [77]. IL-33-responsive B1b cells produce macrophage inflammatory protein-1α (MIP-1α), granulocyte–macrophage colony-stimulating factor (GM-CSF) and VEGF that facilitate the recruitment and activation of monocytes and granulocytes, further contributing to an active immune landscape [21]. Thus, B cells play a more dynamic and supportive role in immune regulation, particularly through their diverse cytokine output, which can bolster anti-tumor immunity.

#### 6.2.3. Role as Antigen-Presenting Cells (APCs) in Initiating Adaptive Immune Responses

B cells can present or cross-present tumor antigens to CD4^+^ and CD8^+^ T cells, supporting tumor-infiltrated T cell proliferation, memory formation and persistence within tumors [81,82]. B cells may therefore function as therapeutic APCs to boost the effectiveness of cancer immunotherapy.

#### 6.2.4. Antibody (Ab)-Secreting Effector Cells in Tumor Immunosurveillance

Abs are generally recognized for their anti-tumor activity and underpin many Ab-based cancer therapies [83,84]. By binding tumor-associated neoantigens, their Fc regions engage Fcγ receptors (FcγR) on immune cells such as NK cells, triggering ADCC, Ab-dependent cellular phagocytosis (ADCP) and complement-dependent cytotoxicity (CDC) (Figure 7B) [85]. Tumor-infiltrating IgG-biased B cell clones and plasma cells have been associated with favorable outcomes in selected cancers, including breast cancer and non-small cell lung cancer (NSCLC) [86,87]. Tumor-associated B cells can differentiate into Ag-specific Ab-secreting plasma cells and produce Abs against tumor Ags, as demonstrated by human papillomavirus (HPV)-specific IgG responses in HPV-positive head and neck cancers [88,89]. Therapeutic strategies targeting intratumoral Tregs may further enhance these TIL-B responses [88]. IgA responses are even more context-dependent. IgA produced by TIL-B cells can contribute to anti-tumor immunity in selected settings, including ovarian cancer possibly via processes including neoantigen-targeted phagocytosis and non-specific transcytosis.

#### 6.2.5. Mediating Immune–Tumor Tissue Interactions

TIL-Bs constitute a substantial fraction (~40%) of tumor-infiltrating lymphocytes and frequently localize within TLSs, where they undergo Ag-driven clonal expansion and affinity maturation [86,90,91,92,93]. Their presence, particularly in breast cancer, is associated with stronger anti-tumor immunity and improved clinical outcomes, highlighting their potential role in tumor immunosurveillance [86,91,92,93].

## 7. Tumor-Infiltrating B Cells (TIL-Bs) and Tertiary Lymphoid Structures (TLSs): Spatial Immunometabolism

TIL-Bs can form organized aggregates with T cells, DCs and stromal elements that resemble secondary lymphoid tissues. Mature TLSs can support Ag presentation, GC-like reactions, affinity maturation and local Ab production. Across several solid tumors, TLS and B cell signatures correlate with response to immune checkpoint blockade and favorable survival, although these associations are not universal [87,91,94,95,96].

The metabolic dimension of TLS biology is an important emerging area. TLSs are spatially organized structures embedded within a heterogeneous tumor and their function is likely to depend on local vascularization, oxygenation, nutrient supply, stromal composition and metabolite gradients. A metabolically permissive TLS may sustain B cell proliferation, GC reactions and Ab production, whereas poorly perfused or highly acidic niches could restrict these processes [97,98]. At present, direct spatial metabolomic measurements of TLS-associated B cells are limited, making this a major research priority rather than an established mechanistic rule.

The prognostic effect of TLS/B cell abundance also varies by tumor type. Favorable associations have been reported in breast cancer, NSCLC and selected gastrointestinal cancers, while pancreatic ductal adenocarcinoma provides an important counterexample in which neoadjuvant chemotherapy has been associated with suppression of the B cell-centered immune landscape [99]. Thus, TLS may be viewed as a functional state whose cellular composition, maturation and metabolic context matter more than simple presence or absence.

## 8. Tumor Type Heterogeneity and the Duality of B Cell Function

An important consideration in interpreting the role of B cells in cancer is the substantial heterogeneity observed among tumor types. B cell infiltration and TLS formation are not uniformly associated with either tumor suppression or tumor progression. Instead, tumor type, stage, inflammatory status, treatment exposure, Ag repertoire, tissue compartment and metabolic context of TME influence B cell behavior. In breast cancer and some lung and gastric tumors, the presence of TLS and clonally expanded IgG-biased TIL-B phenotypes and B cell gene signatures have been associated with enhanced anti-tumor immunity and improved clinical outcomes [86,93]. In NSCLC, intratumoral plasma cells have been linked to response to PD-L1 blockade [100]. By contrast, pancreatic ductal adenocarcinoma can display a B cell-centered immune landscape that is suppressed by neoadjuvant chemotherapy [99].

A similar complexity is evident for Ab isotypes. IgG-producing B cells can support tumor immunity through ADCC, ADCP and complement activation, whereas immune complexes generated by tumor-reactive Abs may, under some circumstances, promote inflammatory and pro-angiogenic responses. IgA-producing B cells have also demonstrated that context-dependent functions may be protective in one tumor and associated with adverse outcomes in another [65,66,67]. These observations suggest that Ab isotype, Ag specificity and the signals driving B cell differentiation are important determinants of biological outcome. Murine B1a/Breg, FasL and GABA studies provide valuable mechanistic hypotheses but cannot automatically be extrapolated to human tumors. The strength of evidence should therefore be considered along three axes: (i) experimental system—human tissue, organoid or mouse model, (ii) functional evidence—correlation versus causal perturbation and (iii) treatment context—untreated disease versus therapy-exposed TME.

Tumor type heterogeneity has direct implications for biomarker development. A high B cell count may be insufficient to predict benefit from immunotherapy unless the phenotype including B cell subset composition, clonality, Ab isotype, spatial organization, metabolic state and interactions with other immune populations is simultaneously characterized. Such multidimensional characterization with composite biomarkers integrating B cell subsets with TLS maturation, metabolic signatures and treatment history may provide a more reliable predictor of treatment response than conventional measures of B cell infiltration alone.

## 9. B Cell Immunometabolism and Therapeutic Resistance

Therapeutic resistance is considered as a dynamic ecological response rather than a single molecular event. B cell states can influence treatment sensitivity through Ag presentation, Ab effector mechanisms, cytokine networks, myeloid cell polarization and organization of TLSs. Metabolic stress may amplify these effects by altering the capacity of B cells and neighboring immune cells to sustain anti-tumor activity [101]. However, evidence that a defined B cell metabolic pathway directly causes resistance to a specific treatment remains limited. Accordingly, the following discussion distinguishes established B cell functions from plausible immunometabolic mechanisms that require experimental validation.

The metabolic characteristics of the TME may influence therapeutic resistance by altering the functional relationship between B cells and other immune populations [102]. Resistance to immune checkpoint inhibitors (ICIs), chemotherapy and radiotherapy is rarely attributable to a single cellular population. Rather, it develops through a network of tumor cell adaptation, impaired Ag presentation, immune suppression, metabolic stress and tissue remodeling. B cells may participate in this network by either supporting effective anti-tumor immunity or reinforcing immune suppression.

In tumors enriched in Bregs, IL-10- and IL-35-mediated suppression can limit the activity of cytotoxic T cells and NK cells and thereby weaken the immune response required for effective tumor control [103]. When combined with hypoxia, nutrient deprivation, lactate accumulation and adenosine-mediated immunosuppression, these B cell-dependent mechanisms may contribute to an immune environment in which checkpoint blockade is less effective [104]. In this context, B cell-mediated resistance is likely to occur primarily through modification of the broader immune network rather than through a direct interaction between B cells and therapeutic agents. Conversely, TLS-associated B cells and clonally expanded TIL-B cells may support treatment efficacy by presenting tumor Ags, producing tumor-reactive Abs and sustaining interactions with CD4^+^ and CD8^+^ T cells [9]. Thus, B cell abundance alone may not be an adequate predictor of treatment response. The phenotype, localization, clonality, Ab isotype and metabolic state of B cells may be more informative determinants of therapeutic outcome.

Metabolic mediators may also influence the response to chemotherapy and radiotherapy. These treatments can alter tumor Ag availability and reshape the immune composition of the TME [105]. In some settings, increased B cell infiltration following treatment has been associated with improved outcome and TLS formation. However, whether treatment-induced metabolic alterations directly reprogram B cell metabolism and determine the subsequent response remains insufficiently understood. The relationship between B cells and cellular therapies, including chimeric antigen receptor T (CAR-T) therapy, is similarly complex. B cell-rich or metabolically suppressive TMEs may indirectly impair cellular therapy by promoting T cell dysfunction and limiting persistence or effector activity. At the same time, B cell-targeting strategies can be therapeutically beneficial in B cell malignancies when the malignant B cell population itself represents the therapeutic target [106]. These observations highlight the importance of distinguishing between B cells as therapeutic targets, B cells as immune regulators, and B cells as components of the metabolically altered TME.

Overall, current evidence supports a model in which metabolic stress can alter B cell phenotype and, through interactions with T cells, NK cells, macrophages and other stromal populations, influence therapeutic sensitivity. However, direct evidence linking specific B cell metabolic pathways to resistance caused by individual treatment modalities remains limited. Future studies should therefore determine whether selective metabolic manipulation of B cell subsets can improve responses to ICIs, chemotherapy, radiotherapy and cellular therapies without eliminating beneficial anti-tumor B cell functions.

### 9.1. Immune Checkpoint Blockade (ICB)

B cell-rich tumors and mature TLSs have been associated with improved responses to ICIs in several tumor types [87,107,108,109]. Mechanistically, TLS-associated B cells can present Ag, support T cell activation and generate tumor-reactive Abs. Conversely, suppressive B cell populations may contribute to an ICI-resistant TME through IL-10/IL-35 production, GABA-mediated macrophage conditioning and reinforcement of regulatory networks. Metabolic signals such as hypoxia, adenosine, PGE2 and lactate can simultaneously impair effector cell function, making it plausible that B cell state and TME metabolism jointly influence checkpoint blockade efficacy. Nevertheless, direct evidence demonstrating that a specific B cell metabolic pathway is sufficient or necessary to mediate ICI resistance, remains scarce. These associations should therefore be interpreted as a mechanistic hypothesis supported by convergent evidence rather than as an established causal pathway.

Therefore, the current literature supports a model in which B cell phenotype is both a marker of the immune ecosystem and a potential contributor to resistance, but prospective studies that manipulate B cell metabolic pathways are required to establish causality.

### 9.2. Chemotherapy

Chemotherapy can reshape the immune landscape through immunogenic cell death, depletion of suppressive populations and changes in Ag availability. Increased B cell or plasma cell infiltration has been associated with favorable outcomes in selected treatment settings [99,100,110,111,112]. However, this relationship is not universal, and pancreatic ductal adenocarcinoma demonstrates that chemotherapy can suppress B cell-centered immunity [99]. Metabolic stress induced by therapy may therefore select for distinct B cell states depending on tumor type and treatment regimen.

### 9.3. Radiotherapy

Radiotherapy can release tumor Ags and alter cytokine and chemokine production, potentially promoting local immune organization. At the same time, radiation-induced hypoxia, tissue injury and inflammatory lipid mediators can generate suppressive niches [113]. B cell responses post-radiotherapy remain less well characterized than T cell and myeloid responses. The possibility that radiation-induced metabolic remodeling changes B cell recruitment, Ag presentation or Ab class switching warrants further investigation.

### 9.4. CAR-T and Cellular Therapies

CAR-T therapy has produced major clinical advances, particularly in B cell malignancies, but efficacy is influenced by tumor burden, Ag escape, T cell fitness and the TME [114,115,116,117,118]. In solid tumors, metabolically hostile environments characterized by hypoxia, nutrient competition and suppressive metabolites can compromise cellular therapy. B cells may influence this setting indirectly through Ag presentation, Ab responses and cytokine networks. Conversely, B cell depletion associated with CD19-directed CAR-T therapy illustrates the therapeutic trade-off between eliminating malignant B cells and perturbing normal B cell immunity. At present, evidence for B cell metabolic pathways as direct determinants of CAR-T resistance is limited and substantially less developed than the evidence for T cell metabolic dysfunction. Accordingly, proposed B cell/TME metabolic coupling should be considered a promising hypothesis and therapeutic opportunity rather than a clinically established resistance mechanism.

## 10. B Cell-Centered Therapeutic Strategies: Opportunities and Limitations

Growing evidence has repositioned B cells from passive components of the TME to potential therapeutic partners in cancer immunotherapy. Tumor Ag stimulation can induce durable, tumor-reactive IgG responses and immune memory, while B cells can support anti-tumor immunity through Ag presentation, cytotoxic T cell activation, Th1 responses and cytokine production [87,99,109,119]. Importantly, increased TIL-Bs, particularly naïve B cells and plasma cells, have been associated with better responses to chemotherapy and improved survival in several cancers [98,99]. Increased TIL-Bs following dabrafenib/trametinib treatment in melanoma and chemotherapy-associated enrichment of inducible co-stimulator ligand on CD19^+^ B (ICOSL^+^ B) cells in breast cancer have also been linked to favorable responses and TLS formation [107,108,109]. Increased CD20^+^ B cell infiltration after neoadjuvant therapy in ovarian cancer further supports the potential of B cell-rich tumors as a therapeutic context [120]. Together, these observations suggest that enhancing beneficial B cell responses and TLS formation may improve treatment efficacy.

Current B cell-centered approaches include monoclonal antibodies (mAbs), B cell depletion strategies and Ab-based immune targeting, while B cells also contribute to the broader immune mechanisms underlying cellular therapies [114,121]. Therapeutic mAbs such as rituximab target TAA and recruit FcγR-expressing effector cells, including NK cells, to promote ADCC [122]. Other Ab-based approaches targeting disialogangloside (GD)2, CD20 and CD22 have demonstrated clinical utility in selected malignancies [85,123]. These strategies highlight the therapeutic potential of exploiting Ab-mediated recognition and Fc-dependent immune effector mechanisms. Targeting specific lymphocyte surface Ags, including CD40 [124], CD20, CD19, CD16A [125] and CD73 [126] can further enhance therapeutic efficacy. In addition, modulating immune checkpoints, preventing specific ligand–receptor interactions, interfering with signaling pathways involving epidermal growth factor receptor (EGFR) and human epidermal growth factor receptor 2 (HER2) [127] and employing other targeted mechanisms may further improve the therapeutic benefits. 

The therapeutic landscape ranges from depletion of malignant or suppressive B cells to activation, engineering or spatial organization of beneficial B cell responses. Established Ab therapies directed against B cell Ags demonstrate the clinical feasibility of targeting this lineage, while emerging approaches seek to exploit B cells as APCs, Ab factories or components of TLS-mediated immunity [107,115,116,127,128,129,130,131,132,133,134,135,136,137,138,139].

**Figure 8:**
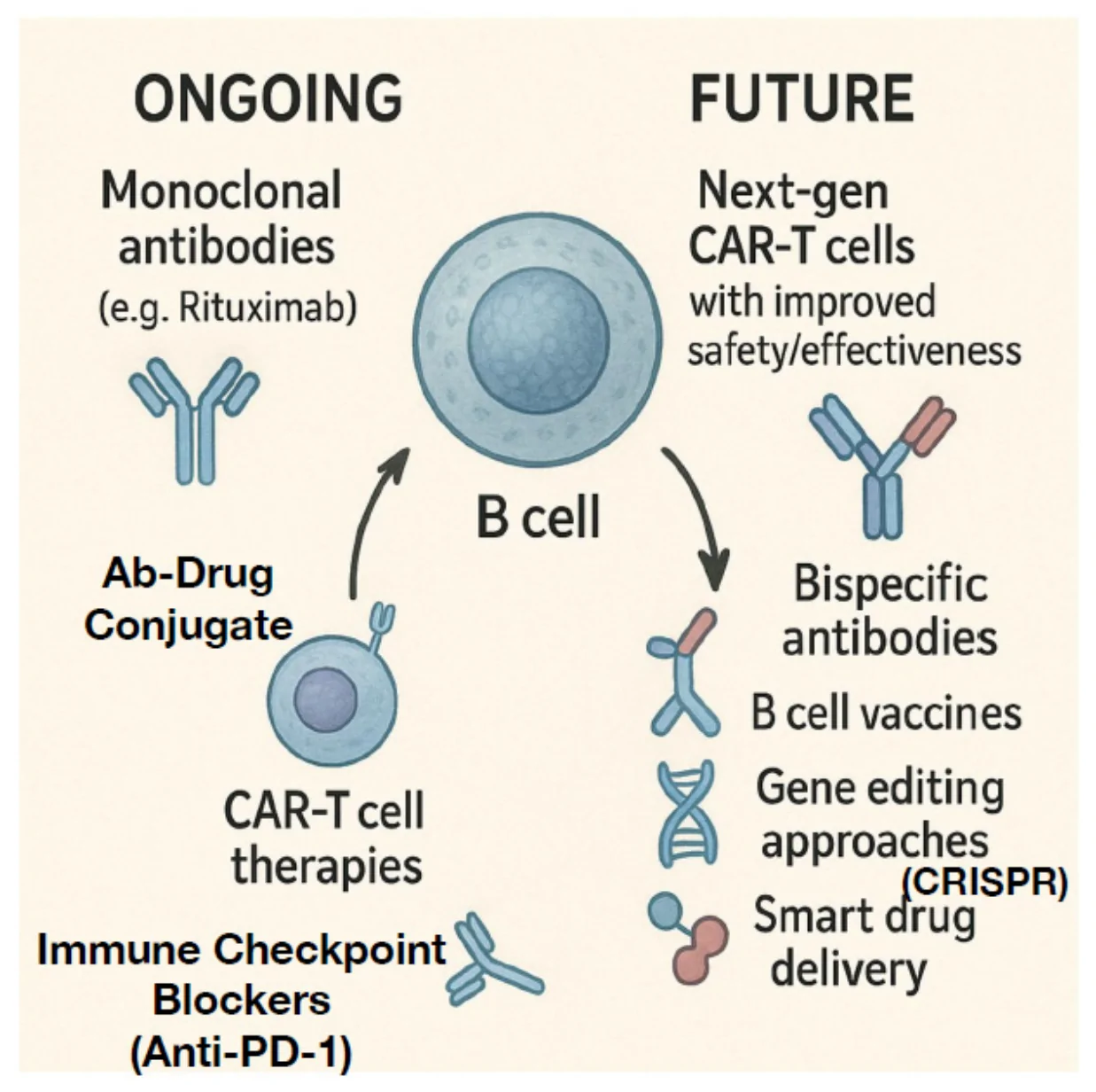
Current and emerging B cell-centered cancer therapies. B cell-targeted therapeutic strategies range from established approaches, including monoclonal antibodies (mAbs), CAR-T cell therapy and B cell depletion, to emerging next-generation interventions designed to harness or selectively reprogram B cell function. These include bispecific Abs, B cell-based vaccines, engineered or include clustered regularly interspaced short palindromic repeats (CRISPR)-modified B cells, Breg modulation, TLS enhancement, nanoparticle/RNA delivery and metabolic targeting, aiming to improve therapeutic efficacy and safety while advancing personalized cancer immunotherapy. The overarching goal is to selectively eliminate detrimental B cell states while preserving or enhancing tumor-reactive B cell responses and durable anti-tumor immunity.

The evolution of B cell-based immunotherapies highlight both current clinical applications and promising future innovations and is depicted in Table 2 and Figure 8. Immune checkpoint inhibitors such as nivolumab, pembrolizumab and ipilimumab can restore anti-tumor T cell activity, while next-generation CAR-T cells aim to improve persistence, safety and resistance to tumor escape [140,141]. Bispecific Abs offer another strategy by physically linking immune effector cells to tumor cells [123]. B cell-based vaccines may promote durable tumor-specific immunity and immune memory [128]. Emerging approaches include clustered regularly interspaced short palindromic repeats (CRISPR)-mediated engineering of immune cells to enhance anti-tumor function and overcome suppressive TMEs [129], together with nanoparticle and other targeted delivery systems designed to improve therapeutic localization while reducing systemic toxicity [130,131]. Together, these future innovations reflect a shift toward more tailored and intelligent B cell-based immunotherapies that aim to revolutionize cancer treatment.

The therapeutic landscape is shifting from broad B cell depletion toward selective exploitation and reprogramming of beneficial B cell functions. A major challenge is the functional heterogeneity of tumor-associated B cells: while TLS-associated and tumor-reactive B cell responses may support treatment, regulatory or immunosuppressive populations can promote tumor progression and therapeutic resistance. Defining B cell phenotype, clonality, spatial organization and metabolic state will therefore be essential for identifying patients most likely to benefit from B cell-centered interventions and for developing safer, more precise combination therapies.

A key implication of the immunometabolic framework is that indiscriminate B cell depletion may not be optimal in solid tumors in which B cell/TLS activity is beneficial. Future strategies should increasingly distinguish malignant B cells, suppressive Bregs and metabolically dysfunctional states from tumor-reactive, IgG-biased or TLS-associated B cells. Candidate approaches include selective Breg modulation, metabolic pathway inhibition, TLS induction, nanoparticle-mediated delivery, engineered B cells and combinations with checkpoint blockade.

## 11. Conclusions and Future Perspectives

B cells are metabolically conditioned and highly heterogeneous components of the tumor immune landscape, with context-dependent rather than uniformly protective or suppressive roles. An important emerging concept is that B cell functions range from Breg- and GABA-associated immunoregulatory activity to Ag presentation, tumor-reactive Ab production, cytotoxicity and TLS organization, with these states shaped by hypoxia, nutrient competition, lactate, adenosine, PGE2 and other metabolic or immunoregulatory signals. These influences may determine whether B cell populations predominantly support anti-tumor immunity through Ag presentation, cytotoxic T cell responses and Ab-mediated tumor recognition or instead reinforce immunosuppression through regulatory cytokines and interactions with suppressive myeloid and lymphoid populations. However, the extent to which specific metabolic pathways causally determine these B cell states in human tumors remains incompletely defined.

The therapeutic significance extends beyond direct B cell targeting. B cell-rich TLS and TIL-B cell populations may enhance responses to immunotherapy in selected tumor types, whereas Bregs and metabolically conditioned suppressive B cell states may contribute to therapeutic resistance. Importantly, the relationship between B cells and treatment outcome is highly context-dependent and B cell abundance alone is unlikely to be a sufficient biomarker. Thus, B cell phenotype, localization and functional state may serve both as a marker of the tumor immune ecosystem and a potential determinant of therapeutic resistance, promoting immune suppression and tumor progression.

Future research should move beyond broad B cell lineage markers towards integrated profiling of B cell phenotype, BCR clonality, Ab isotype, epigenetic and metabolic states using single-cell and spatial approaches. Single-cell transcriptomic, proteomic and metabolic profiling of TIL-Bs should be integrated with spatial analysis to define metabolically distinct B cell states within tumors. Spatial metabolomics may be particularly informative for understanding metabolic gradients within TLS and their relationship with B cell activation, differentiation and Ab production. Longitudinal studies are also needed to determine how chemotherapy, radiotherapy and ICB remodel B cell phenotypes during treatment. Mechanistic perturbation studies targeting pathways such as GABA, adenosine, PGE2, hypoxia and nutrient sensing are needed to establish causality and identify therapeutically feasible pathways. Importantly, findings should be interpreted in the context of tumor type, treatment regimen and species of evidence, with greater emphasis on validation in human tumors. Ultimately, the goal should be selective metabolic and functional reprogramming of B cells rather than their indiscriminate depletion. Defining which B cell states promote treatment sensitivity versus resistance could enable more precise strategies to enhance immunotherapy, chemotherapy, radiotherapy and cellular therapies while preserving beneficial humoral immunity.

The interplay between metabolism and immune regulation in the TME suggests that targeting B cells or modulating their function could offer new therapeutic opportunities. Thus, therapeutic strategies should aim not simply to eliminate B cells but to selectively suppress tumor-promoting B cell functions while preserving or enhancing beneficial effector and TLS-associated populations. Targeting B cell metabolic pathways, regulatory cytokine networks or interactions with suppressive myeloid cells may therefore complement existing immunotherapies. A more precise understanding of B cell immunometabolism could ultimately enable the development of more effective personalized therapeutic strategies that exploit the anti-tumor potential of B cells while limiting their contribution to immune evasion and treatment resistance.

## Figures and Tables

**Table 1:** Context-dependent B cell functions in cancer: evidence, metabolic determinants and therapeutic relevance.

B Cell Population/Function	Pro-/Anti-Tumor Effect	Major Mechanism	Metabolic Cues/TME Influence	Evidence	Translational Relevance
**Bregs**	Pro-tumor	IL-10, IL-35	Hypoxia/adenosine, suppressive TME/nutrient stress	Mainly experimental, human associations	Potential therapeutic target
**B1-like cells**	Pro-tumor in selected models	IL-10	Context-dependent	Mainly murine	Requires human validation
**GABA-producing B cells**	Pro-tumor	IL-10^+^ macrophage, immuno-suppressive macrophage polarization	Metabolic signaling	Experimental	Emerging
**IgA^+^ B cells**	Context-dependent	Context-dependent immune modulation	Mucosal/inflammatory environment	Human, experimental	Context-dependent
**TIL-Bs**	Often anti-tumor	Ag presentation/Ab	TLS/metabolic niche	Human, experimental	Biomarker/therapeutic potential
**TLS B cells**	Often anti-tumor	B-T cooperation/Ab	Local metabolic niche	Human, experimental	ICI response
**IgG^+^ effector B cells**	Often anti-tumor	ADCC/ADCP/CDC	Ag/TME dependent	Human, experimental	Therapeutic potential

Abbreviations: Tertiary lymphoid structure (TLS); Ab-dependent cellular phagocytosis (ADCP); complement-dependent cytotoxicity (CDC).

**Table 2:** B lymphocyte-based cancer therapies—current and future prospects.

Therapeutic Modality	Mechanism/Target	Example	Status/Role	Key Limitation/Opportunity	Reference
**Monoclonal antibodies (mAbs)**	Target tumor/B cell surface Ags for ADCC, CDC, or signaling blockade	Rituximab, Ofatumumab, Obinutuzumab	Approved; target CD20^+^ B cell malignancies	Depletion can remove beneficial B cells, resistance/Ag escape	[132,133]
**Bispecific antibodies (bsAbs) & Bispecific T cell engager (BiTEs)**	Engage T cells/immune effectors with B cell tumor Ags	Blinatumomab (CD3 × CD19)	Approved; B cell acute lymphoblastic leukemia	Toxicity, Ag escape and T cell fitness	[134,135,142]
**Ab–drug conjugates (ADCs)**	Deliver cytotoxic payloads through target Ags to malignant B cells	Brentuximab Vedotin (anti-CD30)	Approved; Hodgkins lymphoma	Target heterogeneity and systemic toxicity	[136]
**CAR-B cell therapy**	Genetically engineered B cells as effectors or APC or Ab-producing cells	Experimental CAR-B cells	Preclinical; could offer better persistence and Ab production than CAR-T	Manufacturing, persistence, trafficking and metabolic fitness	[137,138]
**CAR-T cell therapy against B cell Ags**	Engineered T cells recognize CD19/CD20 or related antigens to kill B cell tumors	Axicabtagene ciloleucel, Tisagenlecleucel	Approved; B cell malignancies	TME suppression, Ag loss, persistence and B cell aplasia	[114,115,116]
**B cell checkpoint blockade/modulation**	Targets immune checkpoints on B cells to reactivate them	Anti-PD-1, Anti-PD-L1, TIGIT, CD22 blockade	Research stage; B cells express PD-1 and other checkpoints in TME	B cell-specific causal benefit remains incompletely established	[139,143]
**B cell-based vaccines**	B cells pulsed or engineered to present tumor Ags	Personalized B cell APC vaccines	Early-stage trials; stimulate both humoral and T cell immunity	May induce pro-tumorigenic Abs	[54,144,145]
**TLS enhancement**	Promotes TLS formation to enhance local B–T cell niches	IL-36γ, Lymphotoxin-β	Preclinical; TLS presence correlates with improved response to ICB in some tumors	TLS maturation and tumor-type dependence	[94,95,96]
**Adoptive transfer of B cells**	Transfer of autologous or allogeneic B cells with tumor-specific activity	Tumor-infiltrating B cells (TIL-B)	Preclinical; potential in melanoma, NSCLC, breast cancer	Better responses to TIL therapy	[45,146]
**Breg inhibition**	Suppresses IL-10^+^ Bregs that dampen anti-tumor immunity	Anti-IL-10R, TLR9 agonists	Experimental; reprogramming tumor-promoting Bregs to effector B cells	Breg heterogeneity and systemic immune effects	[140,146]
**Nanoparticle/RNA delivery to B cells**	Targets B cells or lymphoid niches with Ag/RNA cargo	Lipid nanoparticles, B cell-specific ligands, targeted RNA systems	Preclinical; can program B cells to express anti-tumor Abs	Specificity, biodistribution and metabolic effects	[147,148,149,150]
**Immunomodulatory drugs (IMiDs)**	Enhance B cell activation and anti-tumor Ab production	Lenalidomide, Pomalidomide	Approved in multiple myeloma, hematologic malignancies; modulate B cells and T cells	Pleiotropic mechanisms	[151,152]
**Oncolytic Viruses + B Cells**	Use of viral vectors to stimulate B cells or deliver tumor Ags	Modified measles or vaccinia viruses	Experimental; induce B cell activation or Ag delivery	Potentially convert immunologically cold tumors into B cell/T cell-inflamed tumors	[153,154]
**Plasma cell-directed therapies**	Target long-lived plasma cells in tumor survival pathways	Anti-CD38, Anti-BCMA agents	Approved; effective in multiple myeloma	May remove protective humoral immunity	[155,156]
**CRISPR-engineered B cells**	Genetically programmed B cells to produce therapeutic Abs	CRISPR-Cas9 B cell engineering	Experimental; could offer renewable Ab factories inside patients	Enable highly personalized cellular immunotherapy	[157,158]
**Fc engineering of Abs**	Enhance Fc-mediated effector functions (ADCC, ADCP)	XmAb technology, Fc-optimized mAbs	In development; improved killing of tumor cells via B cell-derived Abs	May increase systemic inflammation, off-target immune-cell depletion or toxicity	[159,160]
**TIL-B**	Harnessing B cells from TLSs in tumors	Adoptive B cell transfer	Future/experimental; emerging concept in solid tumors like melanoma and NSCLC	Patients likely to benefit from immunotherapy or TIL-based approaches	[91,101]
**B cell modulation therapies**	Modulating B cell subsets (e.g., regulatory B cells) to enhance anti-tumor immunity	TLR agonists, IL-21 anti-IL-10R mAbs	Experimental; aims to shift balance towards pro-inflammatory, tumor-fighting B cells	Move toward selective reprogramming rather than depletion	[161,162]
**Checkpoint inhibition (B cell-specific)**	Inhibiting B cell checkpoints (e.g., PD-1 on B cells) to restore function	Anti-PD-1/PD-L1 in B cell-rich tumors	Future/experimental; combined with T cell checkpoint inhibition for synergistic effects	Selectively restore exhausted/dysfunctional B cells while avoiding systemic immune activation	[163,164]
**Metabolic targeting of B cells**	Interrupt suppressive metabolic circuits or restore immune fitness	A2AR inhibition to alleviate adenosine-mediated suppression of plasma cell differentiation	Emerging research area, conceptual; adenosine/PGE2/lactate/GABA pathway modulation	Restore B cell/plasma cell fitness and improve anti-tumor humoral responses	[165,166]

Abbreviations: T cell immunoreceptor with immunoglobulin and immunoreceptor Tyrosine-based inhibition motif {ITIM} domain (TIGIT); Toll-like receptor (TLR); ribonucleic acid (RNA); B cell maturation antigen (BCMA); CRISPR-associated protein 9 (Cas 9); adenosine A2a receptor (A2AR).

## Data Availability

The review article contains only literature-derived information. No new data were created or analyzed in this study. Data sharing is not applicable to this article.
